# Comparative Evaluation of Rotary and Hand File Systems for Optimal Obturation in Primary Molar Root Canal Treatment: A Pediatric Perspective

**DOI:** 10.7759/cureus.78860

**Published:** 2025-02-11

**Authors:** Sumyyia Farooq, Mobeen Akhtar, Mehvish Saleem, Humara Iqbal, Sadiq Amin Ahmed Rana, Muhammad Kashif

**Affiliations:** 1 Operative Dentistry, Nishtar Institute of Dentistry, Multan, PAK; 2 Dental Biomaterials, Bakhtawar Amin Medical and Dental College, Multan, PAK; 3 Operative Dentistry, Multan Medical & Dental College, Multan, PAK; 4 Operative Dentistry, Sadiq Amin Medical and Dental Complex, Multan, PAK; 5 Oral Pathology, Bakhtawar Amin Medical and Dental College, Multan, PAK

**Keywords:** canal cleaning efficiency, hand file system, obturation quality, pediatric endodontics, permanent tooth germ integrity, primary molar, pulpitis management, root canal treatment, rotary file system

## Abstract

Background/objectives

Rotary instrumentation has gained significant importance in the endodontic preparation of primary teeth. Shorter appointment durations improve pediatric patient compliance while maintaining the original root canal anatomy is essential to preserve the integrity of the developing permanent tooth germ. Rotary files facilitate this process by reducing treatment time. This study aimed to compare the obturation quality achieved using hand files versus a rotary file system in the root canal treatment of primary molars.

Materials and methods

A total of 316 patients aged 4-10 years with symptomatic pulpitis (duration ≤7 days) were enrolled. Teeth with sinus tracts or mobility graded 2 or higher were excluded. Patients were randomly assigned into two equal groups: Group A (rotary file system) and Group B (hand files) via sealed, opaque envelopes. Following endodontic treatment, intraoral periapical radiographs were taken using the bisecting angle technique, and obturation quality was evaluated.

Results

The mean ages were 6.73 ± 1.53 years in Group A and 6.78 ± 1.55 years in Group B. Most patients (n = 214, 67.72%) were aged 4-7 years, with a male-to-female ratio of 1:1.4 (130 males, 186 females). Obturation quality was achieved in 111 patients (70.25%) in the rotary file group compared to 74 patients (46.84%) in the hand file group, with a statistically significant difference (p = 0.0001).

Conclusion

The rotary file system demonstrated superior obturation quality in the root canal treatment of primary molars compared to hand files, highlighting its efficacy and potential benefits for pediatric endodontic care.

## Introduction

Endodontic instrumentation and root canal obturation are critical steps in root canal treatment, aiming to remove infected tissue from the canal and seal it against future bacterial contamination [1]. Proper canal shaping is a key determinant of the success of root canal treatment [2]. Endodontic files must maintain intimate contact with canal walls to facilitate effective debridement. This is achieved through careful manipulation of instruments and adherence to biologic principles essential for cleaning and promoting healing [3]. The biological goals include removing necrotic pulp, bacteria, and bacterial toxins via instrumentation and irrigation, followed by filling the sterilized canal with resorbable material [4].

Rotary instrumentation in primary teeth offers several advantages such as uniform preparation and effective debridement of root canals but it also has its limitations [5]. Studies, including Govindaraju et al. [6], have reported that rotary systems reduce the duration of root canal preparation in primary teeth. Nickel-titanium (NiTi) rotary instruments are particularly advantageous, providing consistent canal tapering, improving obturation quality, and minimizing the risk of canal perforation [6,7].

Rotary instrumentation has become particularly relevant for primary teeth, where appointment length is crucial for pediatric patient compliance. Maintaining the original canal anatomy is also essential to preserve the integrity of the permanent tooth germ. Rotary files further enhance patient cooperation by significantly reducing treatment time [7,8].

In a randomized controlled trial (RCT) by Govindaraju et al., 45 children aged 4-8 years (23 girls, 22 boys) requiring pulpectomy in primary mandibular molars were randomly assigned to three groups: Group 1 (manual K files), Group 2 (ProTaper NiTi rotary file system (Dentsply Sirona Inc., Charlotte, North Carolina, United States)), and Group 3 (Mtwo rotary file system (Dentsply Sirona Inc.)) [6]. Periapical radiographs taken using the bisecting angle technique were used to assess obturation quality. Group 1 achieved optimal obturation in 60% of mesial canals and 40% of distal canals, with an overall success rate of 53%. Group 2 achieved 73% and 60% optimal obturation in mesial and distal canals, respectively, with an overall success rate of 69%. Group 3 achieved 60% and 53.3% optimal obturation in mesial and distal canals, respectively, with an overall success rate of 57.78%.

While numerous studies have compared the outcomes of hand files and rotary file systems in the root canal treatment of primary molars, the results have been inconsistent. The purpose of this clinical study is to compare the obturation quality of hand files versus rotary file systems in primary molars. This study not only adds to the existing body of literature but also provides valuable local data on these two techniques. Based on the findings, clinicians can determine the superior technique for routine use in achieving optimal obturation quality for pediatric patients.

## Materials and methods

This was a comparative cross-sectional study conducted in the Outpatient Department of Operative Dentistry, Nishtar Institute of Dentistry, Multan, Pakistan, from October 8, 2021, to April 7, 2022. The study was approved by the Research Evaluation Unit, College of Physicians and Surgeons, Karachi (approval number: CPSP/REU/DSG-2018-102-2426 dated October 8, 2021). Informed consent was obtained from parents or guardians.

Sample size calculation and selection

A sample size of 316 (158 per group) was calculated using a 95% confidence level, 80% study power, and optimal obturation quality rates of 53% for hand files and 69% for rotary file systems [6], following a non-probability consecutive sampling technique. Patients aged 4-10 years of both genders with one tooth symptomatic pulpitis of ≤7 days duration were included. Patients with sinus tracts observed during clinical examination or periapical radiolucency on radiographic examination and patients with grade 2 or higher tooth mobility were excluded.

Data collection

Demographic data, including age, gender, tooth location, disease duration, and procedure duration, were recorded. Patients were randomly assigned into two groups using a lottery method with sealed opaque envelopes: Group A (rotary file system) and Group B (hand files). All treatments were performed by a consultant with at least five years of post-fellowship experience. Normal saline solution was used as canal irrigant. Gutta-percha points matching with final preparation files and endomethasone sealer were used as obturating material in both groups. Following the completion of endodontic treatment, intraoral periapical radiographs were taken using the bisecting angle technique. The obturation quality was assessed by an independent consultant blinded to the group allocation to avoid bias. Data were recorded on a pre-designed proforma.

Statistical analysis

Data were entered and analyzed using IBM SPSS Statistics for Windows, Version 25.0 (2917; IBM Corp., Armonk, New York, United States). Mean ± standard deviation (SD) was calculated for continuous variables such as age, disease duration, and procedure duration. Categorical variables, including gender, residence (rural/urban), tooth location, and optimal obturation quality (yes/no), were presented as frequencies and percentages.

The Chi-square test was used to compare the frequency of optimal obturation quality between the two groups, with a p-value ≤0.05 considered statistically significant. Effect modifiers, including tooth location, disease duration, and procedure duration, were controlled through data stratification. Post-stratification Chi-square testing was performed to assess their effect on obturation quality, withp-value ≤0.05indicating significance.

## Results

Table 1 outlines the demographic and clinical features of 316 subjects divided equally into Group A and Group B (158 participants each). The age distribution shows that the majority of participants (n = 214, 67.72%) belong to the age group of 4-7 years with a mean age of 6.74 ± 1.55 years. There were more girls (n = 186, 58.86%) compared to boys. Most participants reported a duration of 1-4 days (n = 188, 59.49%), with an average of 4.05 ± 1.40 days. The operative procedure duration was predominantly ≤60 minutes for 228 (72.15%) cases, with an average of 50.87 ± 12.82 minutes. Anatomically, mandibular teeth were more frequently involved (n = 193, 61.08%) than maxillary teeth. Additionally, a higher proportion of participants were from urban areas (n = 186, 58.86%).

**Table 1:** Demographic and clinical characteristics of study subjects Values are presented as n (%) or mean ± SD Group A: rotary file system; Group B: hand files

Variables	Group A (n=158)	Group B (n=158)	Total (N=316)
Age (years), n (%)			
4-7	109 (68.99%)	105 (66.46%)	214 (67.72%)
8-10	49 (31.01%)	53 (33.54%)	102 (32.28%)
Mean ± SD	6.73 ± 1.53	6.78 ± 1.55	6.74 ± 1.55
Sex, n (%)			
Male	62 (39.24%)	68 (43.04%)	130 (41.14%)
Female	96 (60.76%)	90 (56.96%)	186 (58.86%)
Duration of disease (days), n (%)			
1-4	95 (60.13%)	93 (58.86%)	188 (59.49%)
5-7	63 (39.87%)	65 (41.14%)	128 (40.51%)
Mean ± SD	3.99 ± 1.43	4.09 ± 1.37	4.05 ± 1.40
Duration of operative procedure (minutes), n (%)			
≤60	118 (74.68)	110 (69.62%)	228 (72.15%)
>60	40 (25.32)	48 (30.38%)	88 (27.85%)
Mean ± SD	50.37 ± 12.73	51.58 ± 12.99	50.87 ± 12.82
Anatomical location of tooth, n (%)			
Mandibular	97 (61.39%)	96 (60.76%)	193 (61.08%)
Maxillary	61 (38.61%)	62 (39.24%)	123 (38.92%)
Residence, n (%)			
Rural	62 (39.24%)	68 (43.04%)	130 (41.14%)
Urban	96 (60.76%)	90 (56.96%)	186 (58.86%)

Table 2 compares the clinical outcomes of Group A and Group B, focusing on the quality of obturation and related variables. Group A demonstrated better clinical outcomes overall. Younger participants (4-7 years) showed significantly better obturation quality than the older group (8-10 years), with p-values of 0.0002 and 0.039, respectively. Gender analysis revealed that girls achieved a higher percentage of optimal obturation quality than boys in both groups (p=0.0002). A shorter disease duration (1-4 days) was also associated with superior outcomes (p=0.002). Furthermore, procedures lasting ≤60 minutes resulted in better obturation quality, with a significant p-value of *0.001*. The anatomical location of teeth also influenced outcomes, with mandibular teeth showing higher success rates compared to maxillary teeth (p=0.003). Overall, Group A achieved a higher percentage of optimal obturation quality (n = 111, 70.25%) than Group B (n = 74, 46.84%), highlighting the impact of demographic and procedural factors on clinical outcomes.

**Table 2:** Comparison of clinical outcome and variables between both treatment groups *p ≤0.05 is considered as significant Group A: rotary file system; Group B: hand files

Variables		Group A (n=158), n (%)		Group B (n=158), n (%)		P value
		Optimal obturation quality		Optimal obturation quality		
		Yes	No	Yes	No	
Age groups (years)	4-7	78 (71.56%)	31 (28.44%)	49 (46.67%)	56 (53.33%)	0.0002*
	8-10	33 (67.35%)	16 (32.65%)	25 (47.17%)	28 (52.83%)	0.039*
Gender	Male	38 (61.29%)	24 (38.71%)	29 (42.65%)	39 (37.35%)	0.034*
	Female	73 (76.04%)	23 (23.96%)	45 (50.0%)	45 (50.0%)	0.0002*
Duration of disease (days)	1-4	76 (80.0%)	19 (20.0%)	55 (59.14%)	38 (40.86%)	0.002*
	5-7	35 (55.56%)	28 (44.44%)	19 (29.23%)	46 (70.77%)	0.003*
Length of operative procedure (minutes)	≤60	85 (72.03%)	33 (27.97%)	50 (45.45%)	60 (54.55%)	0.001*
	>60	26 (65.0%)	14 (35.0%)	24 (50.0%)	24 (50.0%)	0.157
Anatomical location of tooth	Mandibular	69 (71.13%)	28 (28.87%)	48 (50.0%)	48 (50.0%)	0.003*
	Maxillary	42 (68.85%)	19 (31.15%)	26 (41.94%)	36 (58.06%)	0.003*
Overall optimal quality of obturation	Yes	111 (70.25%)		74 (46.84%)		0.0001*
	No	47 (29.75%)		84 (53.16%)		

## Discussion

Successful pulp therapy depends on maintaining an aseptic condition within the root canals through effective cleaning and shaping [9]. However, the biomechanical preparation of root canals in primary teeth can be challenging, especially in the presence of convoluted pulpal anatomy [10]. Ideal obturation plays a critical role in the success of pulp therapy, as it must provide a fluid-impervious seal to prevent bacterial ingress [11]. Several factors influence the quality of obturation, including the adequate filling of obturating material up to the apex and the creation of a three-dimensional seal [12]. One of the most common complications following pulp therapy in children is postoperative pain, which can negatively affect the child’s behavior and overall quality of life [13]. This pain is often a result of apical extrusion of debris during instrumentation, which induces an inflammatory response [14]. The use of hand instruments, though common, is time-consuming and can lead to complications such as apical transportation and ledge formation [15]. To ensure an optimal seal, adequately tapered preparations are essential to ensure that the obturating material reaches the apex of the root canal [16].

The introduction of the Ni-Ti rotary file system by Barr et al. revolutionized root canal instrumentation in primary teeth [17]. Among these, the K3 rotary file system, a third-generation Ni-Ti system, is asymmetric in cross-section and features a positive rake angle and varied pitch, allowing it to efficiently remove dentinal shavings from the canal [18]. The system’s safe-end tip minimizes the risk of perforation during canal instrumentation [19]. An in vitro study by Elmsallati et al. demonstrated that the K3 rotary file system caused minimal alteration to the root canal space during instrumentation [20]. Similarly, a clinical study by Rosa et al. compared the instrumentation time and apical displacement between hand K files and the K3 rotary file system, concluding that the rotary system significantly reduced the instrumentation time [21]. Additionally, Topçuoğlu et al. evaluated postoperative pain levels in primary maxillary molars after root canal instrumentation and found that pain was significantly more intense when hand files were used compared to rotary files [22].

In the present study, the age range of the participants was 4-10 years, with a mean age of 6.74 ± 1.55 years. The majority of patients (67.72%) were between the ages of 4-7 years, with a gender distribution of 130 (41.14%) boys and 186 (58.86%) girls, yielding a male-to-female ratio of 1:1.4. Optimal obturation quality was observed in 74 (46.84%) patients treated with hand files, compared to 111 (70.25%) patients treated with the rotary file system, with a statistically significant difference (p-value = 0.0001). These results align with an RCT by Govindaraju et al., where children aged 4-8 years requiring pulpectomy were randomized into three groups: Group 1 (manual K files), Group 2 (ProTaper NiTi rotary file system), and Group 3 (Mtwo rotary file system) [6]. Optimal obturation rates were significantly higher in the rotary file groups, with the ProTaper system achieving 69% optimal obturation compared to 53% in the manual K file group.

A 2017 survey by Govindaraju et al. among Indian dentists found that 66% of practitioners believed a pediatric-specific rotary file would simplify pulpectomy procedures in primary teeth [23]. Previous studies have also suggested that modifying the length, taper, and tip size of rotary files specifically for pediatric use can further enhance the efficiency and effectiveness of pulpectomy procedures [23,24].

In contrast, a previous in vitro study by Silva et al. showed that profiles with a 0.4 mm diameter did not significantly outperform manual instrumentation in terms of cleaning efficiency. However, rotary instrumentation was still found to be more effective than no instrumentation at all [25]. Similarly, Nazari Moghaddam et al. reported that while rotary flex files did not significantly improve cleaning efficiency compared to manual K files, they did show improved efficiency in cleaning the cervical third of the root canal [26], a finding that contrasted with Silva et al. [25].

Moreover, a meta-analysis by Manchanda et al. which reviewed 13 RCTs comparing rotary and manual instrumentation techniques in primary teeth, found no significant difference in the quality of root canal filling between the two methods [27]. However, rotary instrumentation significantly reduced both instrumentation and canal filling times. Their study also noted that while postoperative pain was not significantly different between the two techniques within the first 12, 24, and 72 hours post treatment, rotary instrumentation was associated with less pain at six and 48 hours post-procedure.

Limitations

Potential limitations of this study include its short-term evaluation of obturation quality without assessing long-term clinical outcomes, reliance on radiographic assessment which may not fully capture three-dimensional obturation quality, and exclusion of teeth with sinus tracts or advanced mobility, potentially biasing results toward less complex cases. An uneven male-to-female ratio could limit generalizability, while patient compliance, a critical factor in pediatric dentistry, was not standardized.

## Conclusions

This study concluded that the quality of obturation achieved with the rotary file system is superior to that of the hand file system in the root canal treatment of primary molars. Based on these findings, we recommend the routine use of the rotary file system for primary molar treatments to consistently achieve optimal obturation quality, thereby enhancing the overall success of root canal treatment in pediatric patients.
